# Immunogenicity and immune-persistence of the CoronaVac or Covilo inactivated COVID-19 Vaccine: a 6-month population-based cohort study

**DOI:** 10.3389/fimmu.2022.939311

**Published:** 2022-08-12

**Authors:** Qianhui Hua, Hangjie Zhang, Pingping Yao, Nani Xu, Yisheng Sun, Hangjing Lu, Fang Xu, Yuting Liao, Juan Yang, Haiyan Mao, Yanjun Zhang, Hanping Zhu, Xiaowei Hu, Huakun Lv, Jianmin Jiang

**Affiliations:** ^1^ School of Medicine, Ningbo University, Ningbo, China; ^2^ Department of Immunization Program, Zhejiang Provincial Center for Disease Control and Prevention, Hangzhou, China; ^3^ Department of Virus Inspection, Zhejiang Provincial Center for Disease Control and Prevention, Hangzhou, China; ^4^ Department of Immunization Program, Xihu District Center for Disease Control and Prevention, Hangzhou, China; ^5^ State Key Laboratory of Molecular Vaccinology and Molecular Diagnostics, National Institute of Diagnostics and Vaccine Development in Infectious Diseases, School of Public Health, Xiamen University, Xiamen, China

**Keywords:** COVID-19, SARS-CoV-2, inactivated vaccine, dynamic changes, immunogenicity, persistence

## Abstract

**Background:**

Owing to the coronavirus disease 2019 (COVID-19) pandemic and the emergency use of different types of COVID-19 vaccines, there is an urgent need to consider the effectiveness and persistence of different COVID-19 vaccines.

**Methods:**

We investigated the immunogenicity of CoronaVac and Covilo, two inactivated vaccines against COVID-19 that each contain inactivated severe acute respiratory syndrome coronavirus 2 (SARS-CoV-2). The levels of neutralizing antibodies to live SARS-CoV-2 and the inhibition rates of neutralizing antibodies to pseudovirus, as well as the immunoglobulin (Ig)G and IgM responses towards the spike (S) and nucleocapsid (N) protein of SARS-CoV-2 at 180 days after two-dose vaccination were detected.

**Results:**

The CoronaVac and Covilo vaccines induced similar antibody responses. Regarding neutralizing antibodies to live SARS-CoV-2, 77.9% of the CoronaVac vaccine recipients and 78.3% of the Covilo vaccine recipients (aged 18–59 years) seroconverted by 28 days after the second vaccine dose. Regarding SARS-CoV-2-specific antibodies, 97.1% of the CoronaVac vaccine recipients and 95.7% of the Covilo vaccine recipients seroconverted by 28 days after the second vaccine dose. The inhibition rates of neutralizing antibody against a pseudovirus of the SARS-CoV-2 Delta variant were significantly lower compared with those against a pseudovirus of wildtype SARS-CoV-2. Associated with participant characteristics and antibody levels, persons in the older age group and with basic disease, especially a chronic respiratory disease, tended to have lower anti-SARS-CoV-2 antibody seroconversion rates.

**Conclusion:**

Antibodies that were elicited by these two inactivated COVID-19 vaccines appeared to wane following their peak after the second vaccine dose, but they persisted at detectable levels through 6 months after the second vaccine dose, and the effectiveness of these antibodies against the Delta variant of SARS-CoV-2 was lower than their effectiveness against wildtype SARS-CoV-2, which suggests that attention must be paid to the protective effectiveness, and its persistence, of COVID-19 vaccines on SARS-CoV-2 variants.

## Introduction

Severe acute respiratory syndrome coronavirus 2 (SARS-CoV-2) has spread worldwide since December 2019, causing the coronavirus disease 2019 (COVID-19) pandemic. Multiple control measures have been taken by the global community to cope with the current pandemic, including wearing a medical mask, maintaining social distancing, performing hand hygiene, and quarantining (1). Even with the recent approval of non-pharmacological interventions, there is still an urgent need for efficient and safe COVID-19 vaccines.

The SARS-CoV-2 RNA genome is approximately 30 kb long and encodes four structural proteins: spike (S) glycoprotein, nucleocapsid (N) protein, membrane (M) protein, and envelope (E) protein (2). The SARS-CoV-2 virus initiates infection in the human body through the binding of its S protein to the host cell receptor angiotensin-converting enzyme 2 (ACE2), which induces neutralizing antibody (NAb) responses and is therefore an important target for vaccine development (3–6). Nucleocapsid protein (NP) is one of the predominantly expressed structural proteins and has high specificity and relatively high sensitivity in the diagnosis of SARS-CoV-2 in the early phase of infection (7, 8). Neutralizing antibody levels have been experimentally shown to be one of the main correlates of protection against SARS-CoV-2 (9). IgG and IgM antibodies that can neutralize the virus by binding to the spike and other membrane proteins and thus preventing infection (10).

COVID-19 vaccines with different designs have been developed and authorized for human use since 2020 to combat this outbreak, focused on five types: RNA vaccine, protein subunit vaccine, inactivated vaccine, non-replicating viral vector vaccine, and DNA vaccine. As of April 15, there were 197 vaccine candidates, 37 approved vaccines, and 10 vaccines that have been granted an Emergency Use Listing (EUL) by World Health Organization (WHO) for COVID-19 worldwide (11). Increasingly more vaccine manufacturers have released the results of phase 3 clinical trials, including those for three inactivated vaccines (CoronaVac, Covilo, WIBP-CorV) that have obtained conditional marketing authorization in China. CoronaVac and Covilo are also included in the WHO emergency use listings. The efficacies of Covilo against symptomatic and severe diseases were 78.1% and 100%, respectively (12). The reported efficacy of CoronaVac varies widely across countries. For symptomatic cases, the reported efficacies of CoronaVac are 83.5%, 65.30%, and 50.7% in Turkey, Indonesia, and Brazil, respectively, and for severe cases, CoronaVac had a reported efficacy of 100% in Brazil (13, 14). In Chile, CoronaVac also showed effectiveness levels of 65.9%, 87.5%, 90.3%, and 86.3% against symptomatic cases, hospitalized cases, cases requiring intensive care unit (ICU) admission, and confirmed death (15).

With the successful development and use of COVID-19 vaccines in some countries, the monitoring of COVID-19 vaccines effectiveness and long-term immunity are still desperately demanded not only to prevent its spread but also to restore social and economic activities *via* generating mass immunization. Here, we aimed to characterize the levels of neutralizing antibodies to live SARS-CoV-2 and the inhibition rates of neutralizing antibodies to pseudovirus, as well as the IgG and IgM responses towards the S and N proteins of SARS-CoV-2, at 180 days after two-dose vaccination with one of two different types of inactivated COVID-19 vaccine, CoronaVac and Covilo.

## Materials and methods

### Study design and participants

Study participants were recruited from the community to assess two-dose regimens of CoronaVac or Covilo. During the period from October to December 2020, the stratified random sampling method was used to select persons aged 18–59 years from the 10 units carrying out COVID-19 vaccination in Xihu District of Hangzhou City, including six streets (towns).

For phase 1, the recruited participants were required to be healthy; individuals with allergies, fever, serious illness, acute or chronic infection, diabetes, hypertension, or another underlying disease and contraindication were excluded. Eligible participants were enrolled to receive two doses of CoronaVac (Cohort 1). After phase 1 recruitment, we considered Covilo vaccine and conducted phase 2 enrollment. For phase 2, participants were recruited to be heathy and complete the two-dose immunization with Covilo (Cohort 2).

### Procedures

**Figure 1:**
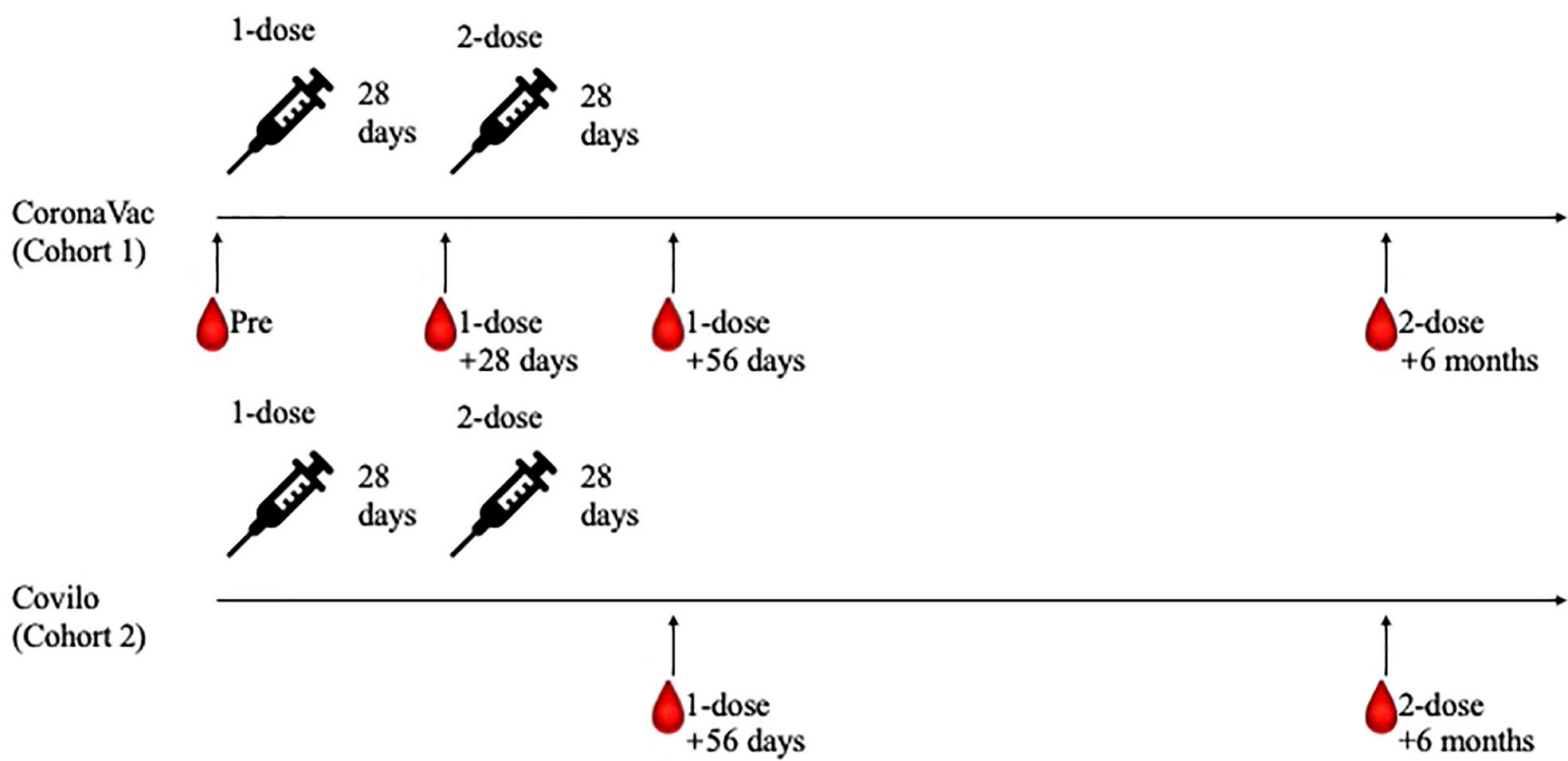
Study processes for the two participant cohorts. Eligible participants were enrolled to receive two doses of CoronaVac on day 0 and day 28 (Cohort 1). After phase 1 recruitment, participants completed the two-dose immunization with Covilo were recruited (Cohort 2). Blood samples were collected and tested at Day 0, Day 28, Day 56 and Day 210 for Cohort 1 participants, while at Day 56 and Day 210 for Cohort 2 participants.

The planned vaccine dosing schedule was to receive doses on day 0 and day 28; participants in Cohort 1 and Cohort 2 all completed their whole course of COVID-19 vaccination within a 7-day window period of the planned schedule. Blood samples were collected and tested at days 0 (baseline, Day 0), 28 (Day 28), and 56 (Day 56) after the first vaccine dose and at 6 months (Day 210) after the second vaccine dose for Cohort 1 participants and at days 56 (Day 56) after the first vaccine dose and at 6 months (Day 210) after the second vaccine dose for Cohort 2 participants (**Figure 1**). These samples were used to investigate any changes in hematology indexes; to determine the levels of neutralizing antibodies against live SARS-CoV-2; to measure the SARS-CoV-2-specific IgG and IgM antibodies levels; and to assess the inhibition rates of pseudovirus for the wildtype (WT) and the Delta (B.1.617.2) variant of SARS-CoV-2.

The COVID-19 vaccines used in this study were two inactivated vaccines (Vero cells), CoronaVac (Sinovac, Beijing, China) and Covilo (Sinopharm, Beijing, China). CoronaVac was prepared using SARS-CoV-2 (CZ02 strain) containing the new coronavirus antigen 600SU after dissociation. Covilo was prepared using the SARS-CoV-2 19nCoV-CDC-Tan-HB02 strain, which contains the new coronavirus *in vitro* relative potency antigen 6.5 U. Both COVID-19 vaccines were administered in doses of 0.5 ml and were purchased from their manufacturers by the CDC of Xihu District through the Zhejiang Provincial CDC and supplied to the inoculation units.

### Plaque reduction neutralization test

The neutralizing antibodies to live SARS-CoV-2 were quantified using a plaque reduction neutralization test (PRNT); serum samples were first heat-inactivated for 30 min at 56°C and then diluted 1:2 in 96-well plates. After the samples were incubated for 1 h at 37°C together with 50% tissue culture infectious dose (TCID_50_) SARS-CoV-2, the serum/virus mixtures were added to VeroE6 cells in another 96-well plate and incubated for 1 h at 37°C. The cytopathic effect (CPE) on VeroE6 cells was analyzed at 3 days post-infection. Neutralization was defined as the absence of 50% CPE compared with virus controls, and the results were calculated by using the Reed-Muench (16) or Spearman-Kärber method (17). The positive cutoff for defining seropositivity for neutralizing antibodies to live SARS-CoV-2 was 1/4, and the neutralizing antibody detection data that were below the cutoff value were included in the analysis as half of the cutoff value.

### Chemiluminescence immunoassay

The levels of IgG and IgM against SARS-CoV-2 S (spike glycoprotein) and N (nucleocapsid) protein in the serum samples were determined using chemiluminescence qualitative kits (iFlash 2019-nCoV, Shenzhen, China) by Chemiluminescence immunoassay (CLIA). Serum samples with SARS-CoV-2 S and N proteins antigen-coated paramagnetic microparticles form a complex, and then an acridinium-ester-labeled ACE2 conjugate is added to competitively combine with the particles, forming another reaction mixture. The analyzer converts a relative light unit (RLU) into an antibody titer (AU/mL) through a two-points calibration curve. An inverse relationship exists between the amount of SARS-CoV-2 NAb in the sample and the RLU detected by the iFlash optical system. According to the manufacturer, a titer ≥10.0 AU/mL is considered positive (or reactive) for both IgM and IgG.

### Pseudovirus-based virus neutralization test

The infection inhibition rate of SARS-CoV-2 neutralizing antibodies was quantified by using the pseudovirus-based virus neutralization test (pVNT). The serum samples, a positive reference, and a negative reference were each diluted 50 times with phosphate-buffered saline (PBS) and combined with 50 µl of pseudovirus diluent per well in a 96-well plate. These mixtures of sample and pseudovirus were incubated at 37°C and 5% CO_2_ for 1 h. Meanwhile, BHK-21-ACE2 cells were collected and used to prepare a cell suspension with a concentration of 2×10^5^/ml; 100 µl of this cell suspension was added to each well of the plate containing the sample/pseudovirus mixtures and the plate was incubated in a 37°C and 5% CO_2_ cell incubator for 48 h. Finally, the number of green fluorescence protein (GFP)-positive cells per well was read with a porous plate imager (Tecan, Shanghai, SparkCyto), and the inhibition rate of neutralizing antibodies in the sample was calculated. The inhibition rate of neutralizing antibody was calculated as (1 - fluorescence value of each well/average virus control value) × 100%.

### Statistical analysis

We used the Pearson χ^2^ test or Fisher’s exact test for the analysis of categorical outcomes. We calculated 95% confidence intervals (CIs) for all categorical outcomes using the Clopper-Pearson method, with the GMT and corresponding 95% CIs based on the standard normal distribution of the log-transformed antibody titers. For Cohort 1 and Cohort 2, the nonparametric test method was used to compare the log-transformed antibody titers (IgG, IgM) and the inhibition rates of SARS-CoV-2 neutralizing antibodies from different timepoints. When the comparison among groups yielded a significant difference, pairwise comparisons were performed. Hypothesis testing was two-sided, and we considered *p*-values of less than 0.05 to indicate a significant difference. We used SPSS (version 18.0) and GraphPad Prism 9 to conduct all analyses.

## Results

### Study participant characteristics

Between October and December 2020, 137 participants (34.3% male participants, 65.7% female participants; 51.1% aged 18–42 years, 48.9% aged 43–59 years; median age: 42 years) were enrolled in the study. 68 and 69 participants were randomly assigned to receive two doses of CoronaVac and Covilo, respectively. The baseline characteristics of the enrolled participants are shown in **Table 1**. Most participants in both the CoronaVac and Covilo groups reported never smoking (86.6% and 91.3%, respectively) and never consuming alcohol (80.9% and 79.7%, respectively). The participants were divided into four subgroups in accordance with the Chinese body mass index (BMI) classifications: underweight (BMI of <18.5 kg/m^2^), normal weight (BMI of 18.5–23.9 kg/m^2^), overweight (BMI of 24–27.9 kg/m^2^), and obesity (BMI of ≥28 kg/m^2^).

**Table 1:** Comparison of baseline characteristics between the two vaccine groups.

	Cohort 1 (n=68)	Cohort 2 (n=69)	*p*
Gender			0.336^a^
Male	26	21	
Female	42	48	
Age			0.105^a^
18-42	40	30	
43-59	29	38	
BMI			0.114
<18.5	3 (4.4)	3 (4.3)	
18.5~23.9	51 (75.0)	39 (56.5)	
24.0~27.9	11 (16.2)	22 (31.9)	
≥28.0	3 (4.4)	5 (7.2)	
Smoke			0.520
Always	6 (8.8)	5 (7.2)	
Sometimes	3 (4.4)	1 (1.4)	
Never	59 (86.6)	63 (91.3)	
Alcohol			1.000
Always	1 (1.5)	1 (1.4)	
Sometimes	12 (17.6)	13 (18.8)	
Never	55 (80.9)	55 (79.7)	
Chronic respiratory diseases			0.619
Yes	1 (1.5)	3 (4.3)	
No	67 (98.5)	66 (95.7)	
Diabetes mellitus			1.000
Yes	0 (0.0)	1 (1.4)	
No	68 (100.0)	68 (98.6)	
Cardiovascular disease			0.208
Yes	1 (1.5)	5 (7.2)	
No	67 (98.5)	64 (92.8)	
Exercise			0.362^a^
Always	14 (20.6)	13 (18.8)	
Sometimes	38 (55.9)	32 (46.4)	
Never	16 (23.5)	24 (34.8)	
Resting			0.291
Always insomnia	2 (2.9)	5 (7.2)	
Sometimes insomnia	16 (23.5)	21 (30.4)	
Regular	50 (73.5)	43 (62.3)	
**Vaccination history**			
Influenza			0.022
Yes	34 (50.0)	46 (66.7)	
No	33 (48.5)	19 (27.5)	
Unclear	1 (1.5)	4 (5.8)	
Hepatitis A/B			0.367
Yes	41 (60.3)	45 (65.2)	
No	21 (30.9)	22 (31.9)	
Unclear	6 (8.8)	2 (2.9)	
Mumps			1.000^a^
Yes	9 (13.2)	10 (14.5)	
No	39 (57.4)	39 (56.6)	
Unclear	20 (29.4)	20 (29.0)	
Rabies			0.329
Yes	9 (13.2)	15 (21.7)	
No	57 (83.8)	50 (72.5)	
Unclear	2 (2.9)	4 (5.8)	
Typhoid			0.016
Yes	1 (1.5)	2 (2.9)	
No	57 (83.8)	44 (63.8)	
Unclear	10 (14.7)	23 (33.3)	
Hemorrhagic fever			0.213
Yes	1 (1.5)	1 (1.4)	
No	57 (83.8)	50 (72.5)	
Unclear	10 (14.7)	18 (26.1)	

The comparison was analyzed by Fisher’s exact test unless it is marked with ^a^ Chi-squared test.

### Neutralizing antibodies to live SARS-CoV-2

**Figure 2:**
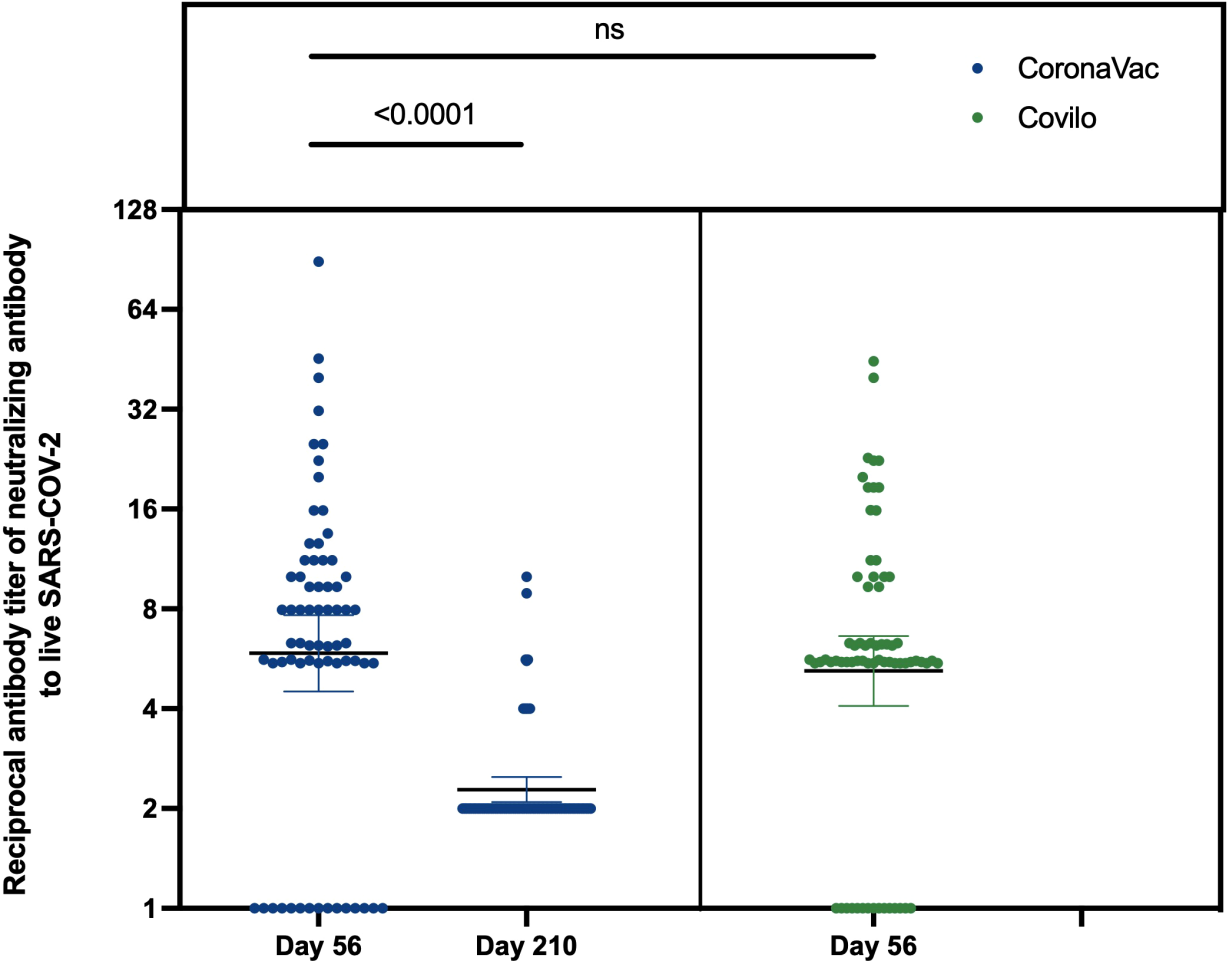
Reciprocal antibody titer of neutralizing antibody to live SARS-CoV-2. The results of neutralizing antibodies to live SARS-CoV-2 on Day 56 and Day 210 in Cohort 1, and Day 56 in Cohort 2. ns: no significance, P>0.05.

The rates seroconversion of neutralizing antibodies to live SARS-CoV-2 were 77.9% (95% CI: 67.8%-88.1%; 53/68 participants) in Cohort 1 (GMT: 10.6 [95% CI: 7.6-13.7]) versus 78.3% (68.3%-88.2%; 54/69 participants) in Cohort 2 (GMT: 8.7 [6.9-10.6]) on Day 56, and were 13.2% (5.0%-21.5%; 9/68 participants) in Cohort 1 (GMT: 4.2 [4.0-4.5]) on Day 210. The difference in levels of neutralizing antibodies between Day 56 and Day 210 in Cohort 1 was statistically significant (*Z* = 5.435, *p* < 0.0001). However, there was no difference in the levels of neutralizing antibodies to live SARS-CoV-2 between Cohort 1 and Cohort 2 on Day 56 (*Z* = 1.326, *p* = 0.185) (**Table 2**, **Figure 2**). Associated with participants’ characteristics, persons with chronic respiratory diseases tended to have lower titers of neutralizing antibodies to live SARS-CoV-2 (*p* = 0.033, **Table 3**).

**Table 2:** Seroconversion rates of antibodies to live SARS-CoV-2, IgG and IgM.

	Seroconversion rates	Antibody levels
Cohort 1		
Neutralizing antibodies to live SARS-CoV-2		
Day 56	53(77.9%; 67.8-88.1)	5.87 (4.6-7.7)
Day 210	9(13.2%; 5.0-21.5)	2.28 (2.1-2.5)
IgG (AU/ml)		
Day 0	0(0.0%)	0.6 (0.4-0.8)
Day 28	19(27.9%; 17.0-38.9)	9.0 (5.9-12.0)
Day 56	66(97.1%; 92.9-101.2)	60.4 (47.3-73.5)
Day 210	17(25.0%; 14.4-35.6)	8.4 (5.8-11.1)
IgM (AU/ml)		
Day 0	0(0.0%)	0.4 (0.3-0.4)
Day 28	7(10.3%; 2.9-17.7)	4.9 (2.9-6.9)
Day 56	9(13.3%; 5.0-21.5)	5.1 (3.2-6.9)
Day 210	0(0.0%)	0.2 (0.1-0.2)
Cohort 2		
Neutralizing antibodies to live SARS-CoV-2		
Day 56	54(78.3%; 68.3-88.2)	5.2 (4.1-6.5)
IgG (AU/ml)		
Day 56	66(95.7%; 90.7-100.6)	55.8 (46.4-65.2)
Day 210	14(20.3%; 10.6-30.0)	6.8 (4.8-8.8)
IgM (AU/ml)		
Day 56	2(2.9%; -1.2-7.0)	1.9 (1.3-2.6)
Day 210	0(0.0%)	0.3 (0.1-0.5)

**Table 3:** Associations between participants’ characteristics and neutralizing antibodies to live SARS-CoV-2.

Variable	Day 56			Day 210		
	Positive	Negative	*p*	Positive	Negative	*p*
Gender						
Male	37 (78.72)	10 (21.28)	0.899 ^a^	1 (3.85)	25 (96.15)	0.138
Female	70 (77.78)	20 (22.22)		8 (19.05)	34 (80.95)	
Age						
18-42	58 (82.86)	12 (17.14)	0.169 ^a^	6 (15.00)	34 (85.00)	0.727
43-59	49 (73.13)	18 (26.87)		3 (10.71)	25 (89.29)	
BMI						
<18.5	4 (66.67)	2 (33.33)	0.773	1 (33.33)	2 (66.67)	0.729
18.5~23.9	69 (76.67)	21 (23.33)		7 (13.73)	44 (86.27)	
24.0~27.9	27 (81.82)	6 (18.18)		1 (9.09)	10 (90.91)	
≥28.0	7 (87.50)	1 (12.50)				
Smoke						
Always	8 (72.73)	3 (27.27)	0.663	0 (0.00)	6 (100.00)	0.730
Sometimes	4 (100.00)	0 (0.00)		0 (0.00)	3 (100.00)	
Never	95 (77.87)	27 (22.13)		9 (15.25)	50 (84.75)	
Alcohol						
Always	2 (100.00)	0 (0.00)	0.310	0 (0.00)	1 (100.00)	1.000
Sometimes	17 (68.00)	8 (32.00)		1 (8.33)	11 (91.67)	
Never	88 (80.88)	22 (20.00)		8 (14.55)	47 (85.45)	
Chronic respiratory diseases						
Yes	1 (25.00)	3 (75.00)	0.033	0 (0.00)	1 (100.00)	1.000
No	106 (79.70)	27 (20.30)		9 (13.43)	58 (86.57)	
Diabetes mellitus						
Yes	1 (100.00)	0 (0.00)	1.000	0 (0.00)	0 (0.00)	
No	106 (77.94)	30 (22.06)		9 (13.24)	59 (86.76)	
Cardiovascular disease						
Yes	3 (50.00)	3 (50.00)	0.119	0 (0.00)	1 (100.00)	1.000
No	104 (79.39)	27 (20.61)		9 (13.43)	58 (86.57)	
Exercise						
Always	21 (77.78)	6 (22.22)	0.709 ^a^	2 (14.29)	12 (85.71)	0.187
Sometimes	53 (75.71)	17 (24.29)		7 (18.42)	31 (81.58)	
Never	33 (82.50)	7 (17.50)		0 (0.00)	16 (100.00)	
Resting						
Always insomnia	5 (71.43)	2 (28.57)	0.252	1 (50.00)	1 (50.00)	0.246
Sometimes insomnia	26 (70.27)	11 (29.73)		1 (6.25)	15 (93.75)	
Regular	76 (81.72)	17 (18.28)		7 (14.00)	43 (86.00)	

The comparison was analyzed by Fisher’s exact test unless it is marked with ^a^ Chi-squared test.

### Anti-SARS-CoV-2 IgG and IgM antibody responses

**Figure 3:**
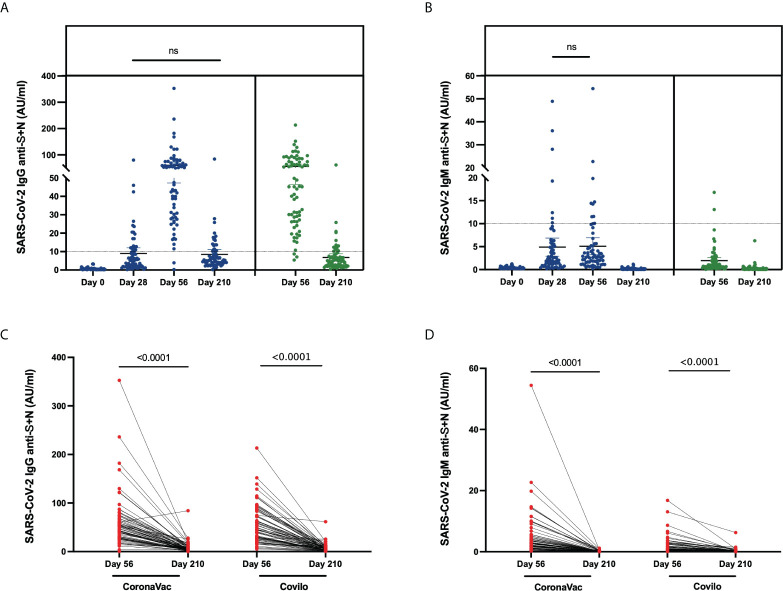
Anti-SARS-CoV-2-specific IgG and IgM levels induced by inactivated COVID-19 vaccines. **(A, B)** Levels of anti-SARS-CoV-2-specific IgG **(A)** and IgM **(B)** in serum samples from CoronaVac-vaccinated participants collected on Day 0, Day 28, Day 56, Day 210 and from Covilo-vaccinated participants on Day 56 and Day 210. **(C, D)** Dynamic changes in anti-SARS-CoV-2-specific IgG **(C)** and IgM **(D)** levels between 28 days (Day 56) and 6 months (Day 210) after the second vaccine dose. ns: no significance, P>0.05.

The levels of IgG and IgM against SARS-CoV-2 S (spike glycoprotein) and N (nucleocapsid protein) protein in serum samples from the vaccinated participants were analyzed. The IgG seroconversion rates in Cohort 1 were 0.0%, 27.9%, 97.1%, and 25.0% on Day 0, Day 28, Day 56 and Day 210, and these rates in Cohort 2 were 95.7% on Day 56 and 20.3% on Day 210 (**Table 2**, **Figure 3A**). Anti-SARS-CoV-2-specific IgG antibodies showed a high antibody seroconversion rate on 28 days after two-dose immunization. The IgM seroconversion rates in Cohort 1were 0.0%, 10.3%, 13.3%, and 0.0% on Day 0, Day 28, Day 56 and Day 210, and these rates in Cohort 2 were 2.9% on Day 56 and 0.0% on Day 210 (**Table 2**, **Figure 3B**).

Through a comparison of the levels at various timepoints, it was found that the titers differed among the different timepoints (*p* < 0.0001). After further pairwise comparison, a significant difference in the level of anti-SARS-CoV-2 IgG was found among all the different timepoints except for Day 28 vs Day 210 (*p* = 0.716) in Cohort 1. It showed that the anti-SARS-CoV-2 IgG antibody increased from Day 0, Day 28, Day 56 and decreased to the similar level of 28 days at six months. The comparison of anti-SARS-CoV-2 IgM levels revealed significant differences among all timepoints except for Day 28 vs Day 56 in Cohort 1 (*p* = 0.807), which showed the level of IgM rose in the short term from Day 0 to Day 28.

From Day 56 to Day 210, the dynamic change in SARS-CoV-2-specific antibody levels (both IgG and IgM) showed a significant downward trend for both Cohort 1 and Cohort 2 (*p* < 0.0001, **Figure 3C, D**). Associated with participants’ characteristics, female participants tended to have a higher IgG-positive rate on Day 210 (*p* = 0.046, **Table 4**), and the IgM seroconversion rate on Day 56 tended to be higher among participants aged 18-42 years than among participants aged 43-59 years (*p* = 0.034, **Table 5**).

**Table 4:** Associations between participants’ characteristics and antibodies to IgG.

Variable	Day 0			Day 28			Day 56			Day 210		
	Positive	Negative	*p*	Positive	Negative	*p*	Positive	Negative	*p*	Positive	Negative	*p*
Gender												
Male	0 (0.00)	26 (100.00)	–	5 (19.23)	21 (80.77)	0.208 ^a^	46 (97.87)	1 (2.13)	0.660	6 (12.77)	41 (87.23)	0.046 ^a^
Female	0 (0.00)	42 (100.00)		14 (33.33)	28 (66.67)		86 (95.56)	4 (4.44)		25 (27.78)	65 (72.22)	
Age												
18-42	0 (0.00)	40 (100.00)	–	14 (35.00)	26 (65.00)	0.121 ^a^	67 (95.71)	3 (4.29)	1.000	20 (28.57)	50 (71.43)	0.089 ^a^
43-59	0 (0.00)	28 (100.00)		5 (17.86)	23 (82.14)		65 (97.01)	2 (2.99)		11 (16.42)	56 (83.58)	
BMI												
<18.5	0 (0.00)	3 (100.00)	–	1 (33.33)	2 (66.67)	0.500	6 (100.00)	0 (0.00)	0.774	2 (33.33)	4 (66.67)	0.030
18.5~23.9	0 (0.00)	51 (100.00)		13 (25.49)	38 (74.51)		87 (96.67)	3 (3.33)		25 (27.78)	65 (72.22)	
24.0~27.9	0 (0.00)	11 (100.00)		3 (27.27)	8 (72.73)		31 (93.94)	2 (6.06)		2 (6.06)	31 (93.94)	
≥28.0	0 (0.00)	3 (100.00)		2 (66.67)	1 (33.33)		8 (100.00)	0 (0.00)		2 (25.00)	6 (75.00)	
Smoke												
Always	0 (0.00)	6 (100.00)	–	1 (16.67)	5 (83.33)	0.579	11 (100.00)	0 (0.00)	1.000	1 (9.09)	10 (90.91)	0.576
Sometimes	0 (0.00)	3 (100.00)		0 (0.00)	3 (100.00)		4 (100.00)	0 (0.00)		1 (25.00)	3 (75.00)	
Never	0 (0.00)	59 (100.00)		18 (30.51)	41 (69.49)		117 (95.90)	5 (4.10)		29 (23.77)	93 (76.23)	
Alcohol												
Always	0 (0.00)	1 (100.00)	–	1 (100.00)	0 (0.00)	0.249	2 (100.00)	0 (0.00)	0.614	0 (0.00)	2 (100.00)	0.879
Sometimes	0 (0.00)	12 (100.00)		2 (16.67)	10 (83.33)		25 (100.00)	0 (0.00)		5 (20.00)	20 (80.00)	
Never	0 (0.00)	55 (100.00)		16 (29.09)	39 (70.91)		105 (95.45)	5 (4.55)		26 (23.64)	84 (76.36)	
Chronic respiratory diseases												
Yes	0 (0.00)	1 (100.00)	–	0 (0.00)	1 (100.00)	1.000	3 (75.00)	1 (25.00)	0.140	2 (50.00)	2 (50.00)	0.220
No	0 (0.00)	67 (100.00)		19 (28.36)	48 (71.64)		129 (96.99)	4 (3.01)		29 (21.80)	104 (78.20)	
Diabetes mellitus												
Yes	0 (0.00)	0 (100.00)	–	0 (0.00)	0 (0.00)	–	1 (100.00)	0 (0.00)	1.000	0 (0.00)	1 (100.00)	1.000
No	0 (0.00)	68 (100.00)		19 (27.94)	49 (72.06)		131 (96.32)	5 (3.68)		31 (22.79)	105 (77.21)	
Cardiovascular disease												
Yes	0 (0.00)	1 (100.00)	–	0 (0.00)	1 (100.00)	1.000	5 (83.33)	1 (16.67)	0.203	2 (33.33)	4 (66.67)	0.618
No	0 (0.00)	67 (100.00)		19 (28.36)	48 (71.64)		127 (96.95)	4 (3.05)		29 (22.14)	102 (77.86)	
Exercise												
Always	0 (0.00)	14 (100.00)	–	5 (35.71)	9 (64.29)	0.335	25 (92.59)	2 (7.41)	0.221	5 (18.52)	22 (81.48)	0.822 ^a^
Sometimes	0 (0.00)	38 (100.00)		8 (21.05)	30 (78.95)		69 (98.57)	1 (1.43)		16 (22.86)	54 (77.14)	
Never	0 (0.00)	16 (100.00)		6 (37.50)	10 (62.50)		38 (95.00)	2 (5.00)		10 (25.00)	30 (75.00)	
Resting												
Always insomnia	0 (0.00)	2 (100.00)	–	1 (50.00)	1 (50.00)	0.654	7 (100.00)	0 (0.00)	1.000	3 (42.86)	4 (57.14)	0.191
Sometimes insomnia	0 (0.00)	16 (100.00)		5 (31.25)	11 (68.75)		36 (97.30)	1 (2.70)		10 (27.03)	27 (72.97)	
Regular	0 (0.00)	50 (100.00)		13 (26.00)	37 (74.00)		89 (95.70)	4 (4.30)		18 (19.35)	75 (80.65)	

The comparison was analyzed by Fisher’s exact test unless it is marked with ^a^ Chi-squared test.

**Table 5:** Associations between participants’ characteristics and antibodies to IgM.

Variable	Day 0			Day 28			Day 56			Day 210		
	Positive	Negative	*p*	Positive	Negative	*p*	Positive	Negative	*p*	Positive	Negative	*p*
Gender												
Male	0 (0.00)	26 (100.00)	–	2 (7.79)	24 (92.31)	0.700	4 (8.51)	43 (91.49)	1.000	0 (0.00)	47 (100.00)	–
Female	0 (0.00)	42 (100.00)		5 (11.90)	37 (88.10)		7 (7.78)	83 (92.22)		0 (0.00)	90 (100.00)	
Age												
18-42	0 (0.00)	40 (100.00)	–	5 (12.50)	35 (87.50)	0.691	9 (12.86)	61 (87.14)	0.034 ^a^	0 (0.00)	70 (100.00)	–
43-59	0 (0.00)	28 (100.00)		2 (7.14)	26 (92.86)		2 (2.99)	65 (97.01)		0 (0.00)	67 (100.00)	
BMI												
<18.5	0 (0.00)	3 (100.00)	–	1 (33.33)	2 (66.67)	0.272	1 (16.67)	5 (83.33)	0.431	0 (0.00)	6 (100.00)	–
18.5~23.9	0 (0.00)	51 (100.00)		4 (7.84)	47 (92.16)		6 (6.67)	84 (93.33)		0 (0.00)	90 (100.00)	
24.0~27.9	0 (0.00)	11 (100.00)		2 (18.18)	9 (81.82)		3 (9.09)	30 (90.91)		0 (0.00)	33 (100.00)	
≥28.0	0 (0.00)	3 (100.00)		0 (0.00)	3 (100.00)		1 (12.50)	7 (87.50)		0 (0.00)	8 (100.00)	
Smoke												
Always	0 (0.00)	6 (100.00)	–	1 (16.67)	5 (83.33)	0.648	1 (9.09)	10 (90.91)	1.000	0 (0.00)	11 (100.00)	–
Sometimes	0 (0.00)	3 (100.00)		0 (0.00)	3 (100.00)		0 (0.00)	4 (100.00)		0 (0.00)	4 (100.00)	
Never	0 (0.00)	59 (100.00)		6 (10.17)	53 (89.83)		10 (8.20)	112 (91.80)		0 (0.00)	122 (100.00)	
Alcohol												
Always	0 (0.00)	1 (100.00)	–	0 (0.00)	1 (100.00)	0.641	0 (0.00)	2 (100.00)	0.515	0 (0.00)	2 (100.00)	–
Sometimes	0 (0.00)	12 (100.00)		2 (16.67)	10 (83.33)		3 (12.00)	22 (88.00)		0 (0.00)	25 (100.00)	
Never	0 (0.00)	55 (100.00)		5 (9.09)	50 (90.91)		8 (7.27)	102 (92.73)		0 (0.00)	110 (100.00)	
Chronic respiratory diseases												
Yes	0 (0.00)	1 (100.00)	–	0 (0.00)	1 (100.00)	1.000	0 (0.00)	4 (100.00)	1.0000	0 (0.00)	4 (100.00)	–
No	0 (0.00)	67 (100.00)		7 (10.45)	60 (89.55)		11 (8.27)	122 (91.73)		0 (0.00)	133 (100.00)	
Diabetes mellitus												
Yes	0 (0.00)	0 (100.00)	–	0 (0.00)	0 (0.00)	–	0 (0.00)	1 (100.00)	1.000	0 (0.00)	1 (100.00)	–
No	0 (0.00)	68 (100.00)		7 (10.29)	61 (89.71)		11 (8.09)	125 (91.91)		0 (0.00)	136 (100.00)	
Cardiovascular disease												
Yes	0 (0.00)	1 (100.00)	–	0 (0.00)	1 (100.00)	1.000	0 (0.00)	6 (100.00)	1.000	0 (0.00)	6 (100.00)	–
No	0 (0.00)	67 (100.00)		7 (10.45)	60 (89.55)		11 (8.40)	120 (91.60)		0 (0.00)	131 (100.00)	
Exercise												
Always	0 (0.00)	14 (100.00)	–	1 (7.14)	13 (92.86)	0.234	0 (0.00)	27 (100.00)	0.093	0 (0.00)	27 (100.00)	–
Sometimes	0 (0.00)	38 (100.00)		6 (15.79)	32 (84.21)		9 (12.86)	61 (87.14)		0 (0.00)	70 (100.00)	
Never	0 (0.00)	16 (100.00)		0 (0.00)	16 (100.00)		2 (5.00)	38 (95.00)		0 (0.00)	40 (100.00)	
Resting												
Always insomnia	0 (0.00)	2 (100.00)	–	1 (50.00)	1 (50.00)	0.239	0 (0.00)	7 (100.00)	0.851	0 (0.00)	7 (100.00)	–
Sometimes insomnia	0 (0.00)	16 (100.00)		1 (6.25)	15 (93.75)		2 (5.41)	35 (94.59)		0 (0.00)	37 (100.00)	
Regular	0 (0.00)	50 (100.00)		5 (10.00)	45 (90.00)		9 (9.68)	84 (90.32)		0 (0.00)	93 (100.00)	

The comparison was analyzed by Fisher’s exact test unless it is marked with ^a^ Chi-squared test.

### The inhibition rate of neutralizing antibody against pseudovirus

**Figure 4:**
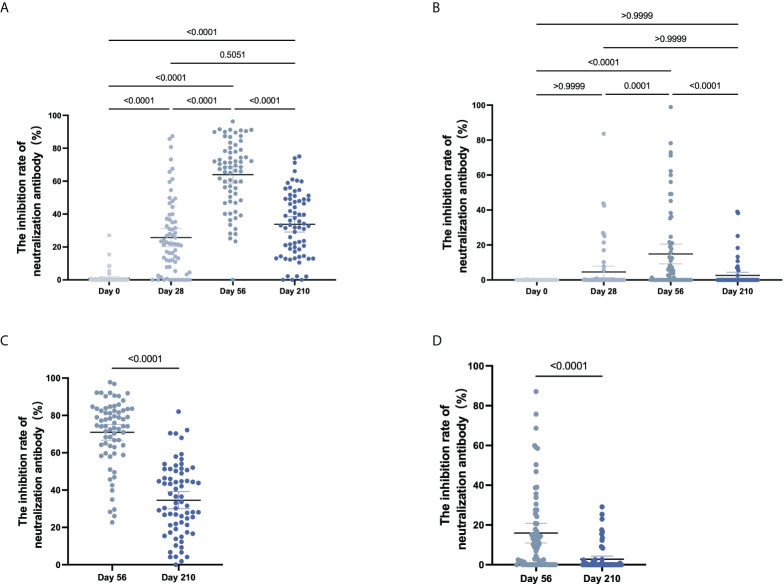
The inhibition rate of neutralizing antibody to pseudovirus. **(A–D)** For CoronaVac-vaccinated **(A, B)** and Covilo-vaccinated participants **(C, D),** the infection inhibition rates of neutralizing antibody to pseudovirus for WT SARS-CoV-2 **(A, C)** or for the Delta variant of SARS-CoV-2 **(B, D)**.

The inhibition rates of neutralizing antibodies against the WT and Delta variant of SARS-CoV-2 after COVID-19 vaccination were shown in **Figure 4**. For Cohort 1, the inhibition rates to pseudovirus for the WT of SARS-CoV-2 were 1.0% (0.2%-2.1%) on Day 0, 25.7% (20.7%-31.2%) on Day 28, 63.9% (59.2%-69.1%) on Day 56, and 33.8% (29.3%-38.3%) on Day 210 (**Figure 4A**). Significant differences in the inhibition rates were found between all timepoints (*p* < 0.001). For the Delta variant of the SARS-CoV-2, inhibition rates of 0.0% on Day 0, 4.6% (1.6%-8.2%) on Day 28, 14.8% (9.7%-20.7%) on Day 56, and 2.6% (0.8%-4.6%) on Day 210 (**Figure 4B**). By pairwise comparison at different time points, Day 56 is statistically different (*p* < 0.001) and no other comparisons reach significance (Day 0 vs Day 28, *p* = 0.0635; Day 0 vs Day 210, *p* = 0.0836; Day 28 vs Day 56, *p* = 0.4428).

**Figure 5:**
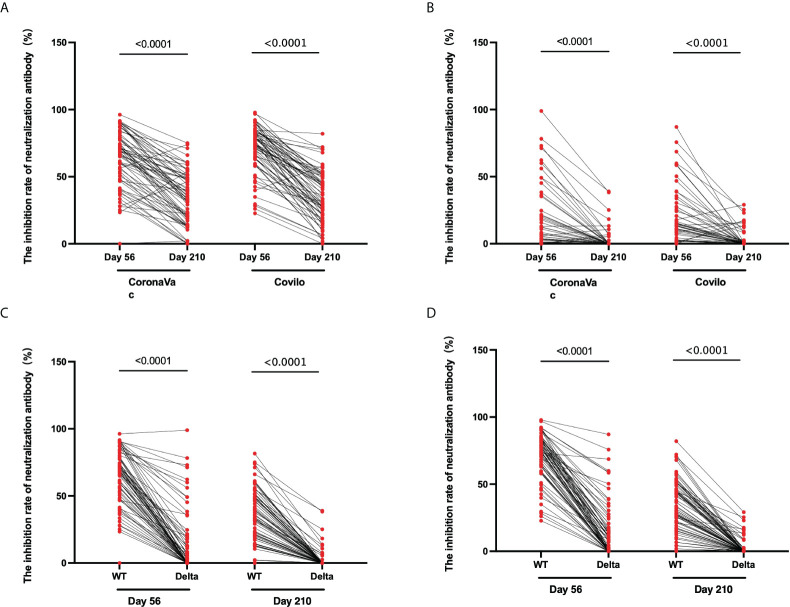
Dynamic changes in the inhibition rates of neutralizing antibody to pseudovirus. **(A, B)** Dynamic changes in infection inhibition rates of neutralizing antibody to pseudovirus against the WT of SARS-CoV-2 **(A)** or the Delta variant of SARS-CoV-2 **(B)** on Day 56 and Day 210 in samples from CoronaVac- and Covilo-vaccinated participants. **(C, D)** Comparison of inhibition rates against pseudovirus for the WT and the Delta variant of SARS-CoV-2 on Day 56 and Day 210 for CoronaVac-vaccinated **(C)** or Covilo-vaccinated **(D)** participants.

For Cohort 2, the inhibition rates of neutralizing antibodies to pseudovirus for the WT of SARS-CoV-2 were 71.0% (66.6%-75.1%) on Day 56 and 34.6% (30.0%-39.0%) on Day 210 (*p* < 0.0001), and those against pseudovirus for the Delta variant of SARS-CoV-2 were 15.9% (11.6%-20.9%) on Day 56 and 2.8% (1.3%-4.4%) on Day 210 (*p* < 0.0001) (**Figure 4C, D**). There was an obvious downward trend in the dynamic changes of inhibition rate (Day 56 vs Day 210) following vaccination with CoronaVac (*p* < 0.0001) or Covilo (*p* < 0.0001), against both the WT SARS-CoV-2 (**Figure 5A**) and the Delta variant of SARS-CoV-2 (**Figure 5B**).

Regarding the infection inhibition rates against pseudoviruses of the WT and the Delta variant of SARS-CoV-2 on Day 56 and Day 210, there were significant decreases in Cohort 1(*p*_1_ < 0.0001, *p*_2_ < 0.0001; **Figure 5C**) and in Cohort 2 (*p*_1_ < 0.0001, *p*_2_ < 0.0001; **Figure 5D**). The infection inhibition rate of neutralizing antibodies against the Delta variant of SARS-CoV-2 was significantly lower than that for neutralizing antibodies against the WT of SARS-CoV-2.

### Correlation among levels of anti-SARS-CoV-2 IgG, IgM, inhibition rates of neutralizing antibodies to pseudovirus, with levels of neutralizing antibodies to live SARS-CoV-2

**Figure 6:**
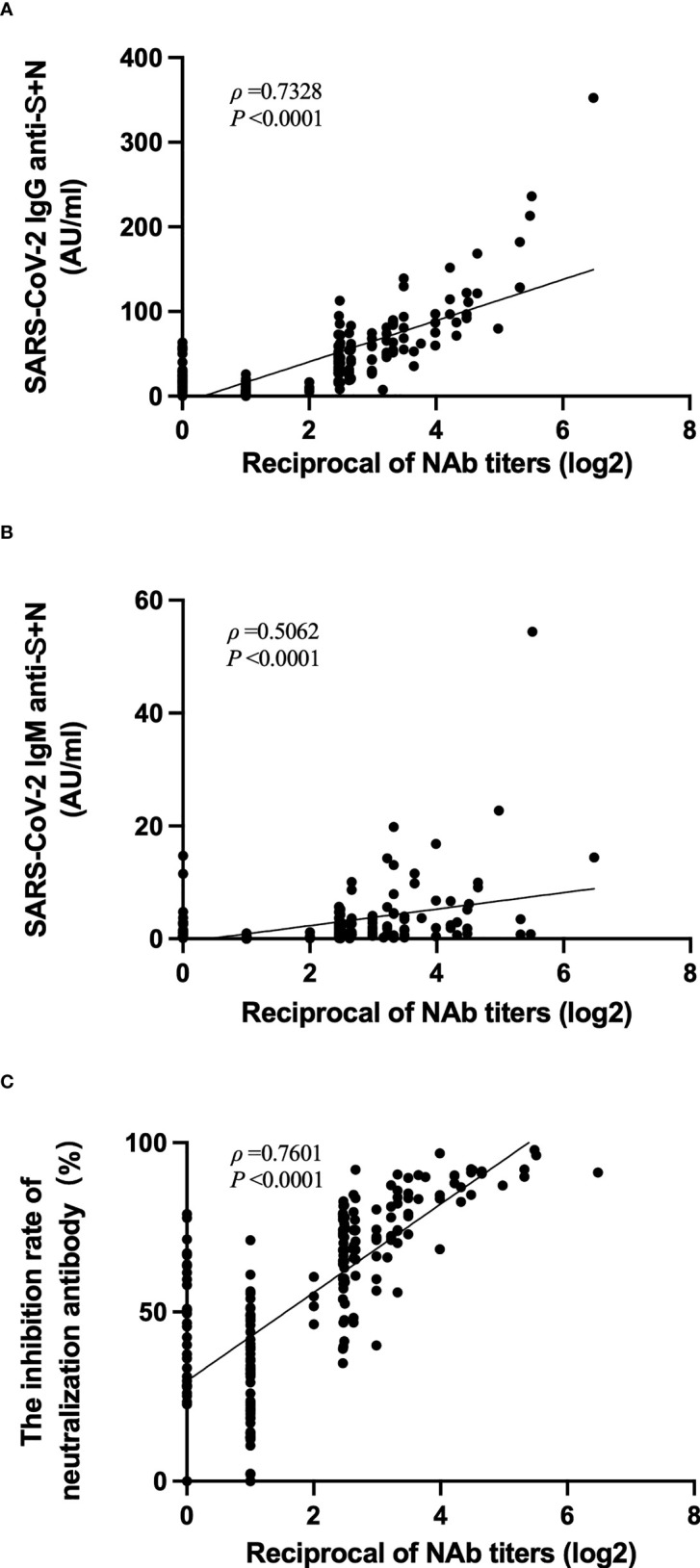
Correlation among levels of anti-SARS-CoV-2 IgG, IgM, inhibition rates of neutralizing antibodies to pseudovirus, with levels of neutralizing antibodies to live SARS-CoV-2. **(A–C)** Correlation of anti-SARS-CoV-2 IgG levels **(A)**, anti-SARS-CoV-2 IgM levels **(B)**, or inhibition rates of neutralizing antibodies to pseudovirus **(C)** with the levels of neutralizing antibodies to live SARS-CoV-2.

The correlation among titers of neutralizing antibody to live SARS-CoV-2 with other tests in the same period was analyzed. The titers of neutralizing antibodies to live SARS-CoV-2 did not correlate well with the anti-SARS-CoV-2 IgM titers (*ρ* = 0.5062, **Figure 6B**). However, these neutralizing antibody titers strongly correlated with the anti-SARS-CoV-2 IgG titers (*ρ* = 0.7328, **Figure 6A**) and with the inhibition rates of neutralizing antibodies to pseudovirus (*ρ* = 0.7601, **Figure 6C**).

## Discussion

The dynamic changes in the levels of neutralizing antibodies to live SARS-CoV-2, levels of anti-SARS-CoV-2 IgG and IgM, and the inhibition rates of neutralizing antibodies to pseudovirus demonstrate that the induced antibody levels did not significantly increase until after the second dose of COVID-19 vaccine. It is well known that neutralizing antibodies targeting the virus play an important role in virus clearance and recovery from viral infection (18, 19).

As many studies have already reported, the neutralization ability of neutralizing antibodies in SARS-CoV-2 convalescent individuals varies from individual to individual (20). In this study, the immune responses induced by the CoronaVac and Covilo vaccines were similar. We found that 77.9% of the vaccine recipients in Cohort 1 and 78.3% of the vaccine recipients in Cohort 2 (aged 18–59 years) seroconverted by 28 days after the second vaccine dose (Day 56). For Cohort 1, the seroconversion rate of the vaccine recipients declined after the second vaccine dose to 13.2% at Day 210.

The titers of SARS-CoV-2 antibody measured indicates that most antibodies generated against S protein and N protein belonged to subtype IgG; there were only low levels of antibodies with subtype IgM (21). This is not surprising; IgG antibody is typically produced for several weeks and lasts for a long time (more than 13 weeks), whereas IgM antibody is transiently expressed (22). This pattern was reproduced here: specific-IgG antibody responses peaked at 28 days after the second vaccine dose and decreased to a low level by 6 months post-vaccination. As for IgM, there was no significant difference in the antibody titers between 28 days after the first vaccine dose and 28 days after the second vaccine dose, and the titers decreased at six months post-vaccination. Similar finding was reported evaluating the IgG response induced a robust antibody response that wanes significantly over time, which showed the seropositivity were 99.8% and 97.9% at 30 days and 6 months after the second dose of CoronaVac (23). The titers of SARS-CoV-2-specific antibodies and neutralizing antibody showed the same trends, in that the titers decreased from 28 days to 6 months after the second vaccine dose.

The neutralizing antibodies induced by the two inactivated COVID-19 vaccines tested here were able to neutralize both the WT and Delta SARS-CoV-2 variants. The inhibition rates of neutralizing antibody to pseudovirus revealed that the neutralizing responses peaked at 28 days after the second vaccine dose (Day 56) and decreased significantly by 6 months after the second vaccine dose (Day 210), mirroring the patterns observed for the levels of neutralizing antibody against live SARS-CoV-2 and of SARS-CoV-2-specific antibodies. Our research showed the similar trend with the results of Cheng ZJ et (24). Compared with the high inhibition rate against WT SARS-CoV-2, the inhibition rate against the SARS-CoV-2 Delta variant was significantly lower, indicating that the neutralization ability against the Delta variant was significantly weaker than that against the WT virus. Interestingly, the SARS-CoV-2 Delta variant was able to escape from neutralizing antibodies from some COVID-19 convalescent sera or was less sensitive to neutralization by these antibodies (25). Variant S proteins promote virus infectivity through enhanced viral entry and membrane fusion, and this may play an important role in the increased transmissibility of the SARS-CoV-2 Delta variant (26). Another explanation for the increased transmission of the Delta variant of SARS-CoV-2 may be an increased ability of the virus to escape the immune system (27). Consistent with this idea, recent studies have reported that some COVID-19 vaccines had lower neutralization effectiveness against the Delta variant; the effectiveness after two doses of BNT162b2 vaccine was 88.0% (95% CI: 85.3–90.1) among persons infected with the Delta variant, and the effectiveness after two doses of ChAdOx1 nCoV-19 vaccine was 67.0% (95% CI: 61.3–71.8) among persons infected with the Delta variant (28, 29).

The presence of neutralizing antibodies is considered a functional correlate of immunity and considered important for viral neutralization and viral clearance (30). Therefore, as part of the validation of new serological tests, the comparison with virus-neutralizing test was very important (31). In our research, correlation among levels of neutralizing antibodies to live SARS-CoV-2 with levels of anti-SARS-CoV-2 IgG or the inhibition rates of neutralizing antibodies to pseudovirus were relatively strong, however, the titers of neutralizing antibodies to live SARS-CoV-2 did not correlate well with the anti-SARS-CoV-2 IgM titers. This might be related to the rapid attenuation of IgM, as the relative contribution of IgG to neutralizing antibody increased and that of IgM further decreased over time (32). Compared to others results, our findings presented weaker response to the inactivated vaccines, which closely related to the period of blood collection and detection methods, as the threshold of protection for antibody titers against COVID‐19 remains unknown.

In this study, three measurements were used to investigate the antibody responses of SARS-CoV-2 generated by two doses of inactivated COVID-19 vaccine: the titers of neutralizing antibodies to live SARS-CoV-2, the titers of neutralizing antibodies to pseudovirus, and the titers of IgG and IgM specific for the S protein and N protein of SARS-CoV-2. All of the antibody titers showed the same patterns; antibodies that were elicited by either of the two inactivated COVID-19 vaccines appeared to wane rapidly following their peak after the second vaccine dose, but they persisted in detectable levels through 6 months after the second vaccine dose, which is consistent with previous results for the mRNA-1273 vaccine (33), BNT161b2 vaccine (34), ChAdOx1 nCoV-19 vaccine (35), CoronaVac vaccine (23) and Covilo vaccine (24). Regarding participant characteristics, persons belonging to the older age group and with an underlying disease, especially a chronic respiratory disease, tended to have lower anti-SARS-CoV-2 antibody seroconversion rates.

There are some limitations in this study. Firstly, we reported only antibody responses for healthy adults, and our study did not include individuals from populations considered to be particularly susceptible to COVID-19, such as those with comorbidities, older individuals (aged ≥60 years), children or adolescents, pregnant or lactating women, and immunocompromised persons. Secondly, we did not assess the T-cell responses in this study. Finally, with the emergence of increasingly more SARS-CoV-2 variants, especially the recent pandemic Omicron variant, it is necessary to continue determining the effectiveness of existing COVID-19 vaccines on the new virus variants as well as the persistence of vaccine effectiveness.

In summary, the two inactivated COVID-19 vaccines CoronaVac and Covilo induced strong humoral responses against SARS-CoV-2; however, the vaccine-induced immune response against the Delta variant of SARS-CoV-2 was weaker than that induced against the WT, which suggests that close attention must be paid to the protective effect of COVID-19 vaccines against SARS-CoV-2 variants. Appropriate COVID-19 immunization procedures should be studied, so that an immune barrier can be gradually established in the population by inoculating people with a COVID-19 vaccine in an orderly manner, thus lowering the prevalence of COVID-19 and restoring our social economy and the normal ways of life as soon as possible.

## Data availability statement

The raw data supporting the conclusions of this article will be made available by the authors, without undue reservation.

## Ethics statement

The studies involving human participants were reviewed and approved by the Ethics Committee of Hangzhou Xihu District Center for Disease Control and Prevention. The patients/participants provided their written informed consent to participate in this study.

## Author contributions

XH, HKL and JJ designed the study. QH, HJZ, YL, JY and YZ analyzed the data, interpreted the results, produced the figures, and prepared the manuscript. QH, NX and HM contributed to the manuscript writing. PY, YS, HJL, FX and HPZ performed the samples detection. HJZ, XH, HKL and JJ supervised the project and revised the manuscript. All authors contributed to writing the manuscript and approved the final manuscript.

## Funding

This work was supported by the Key Research and Development Program of Zhejiang Province (2021C03200); the Key Program of Health Commission of Zhejiang Province/Science Foundation of National Health Commission (WKJ-ZJ-2221); Hangzhou Science and technology Development Research Project(20201203B27).

## Acknowledgments

We would like to thank the staff from the local CDCs and the local designated hospitals for their help with the field survey.

## Conflict of interest

The authors declare that the research was conducted in the absence of any commercial or financial relationships that could be construed as a potential conflict of interest.

## Publisher’s note

All claims expressed in this article are solely those of the authors and do not necessarily represent those of their affiliated organizations, or those of the publisher, the editors and the reviewers. Any product that may be evaluated in this article, or claim that may be made by its manufacturer, is not guaranteed or endorsed by the publisher.
